# Municipal structural adjustment: For an institutional analysis of global development finance

**DOI:** 10.1177/0308518X251349100

**Published:** 2025-08-21

**Authors:** Hanna Hilbrandt

**Affiliations:** Department of Geography, University of Zurich, Zurich, Switzerland

**Keywords:** Development finance, Mexico, critical insitutionalism, structural adjustment, municipal governance

## Abstract

In this paper I examine climate-aligned development finance initiatives (DFIs) that foster private-sector investment in ‘sustainable’ urban infrastructures. I am concerned with the ways in which they work to create the institutional preconditions for such investment. I develop the notion of Municipal Structural Adjustment (MSA) as a lens for examining the fields of intervention and modes of policy-making through which these initiatives seek to reform municipalities’ governance structures, innovate planning tools and introduce financial instruments. Empirically, this paper focuses on initiatives that get implemented in Mexican municipalities. It builds on a historical exploration of the ways in which development interventions have addressed municipal-scale institutions, as well as on expert interviews with development officers, DFI staff, municipal officials, planners and consultants to analyse these actors’ efforts to reform urban bureaucracies. I delineate three interrelated modalities of institutional adjustment that I take to characterise MSA: (1) the superimposition of DFIs’ planning schemes with government legislation and their gradual institutionalisation; (2) the burial of decisions in the development of infrastructure deals and (3) the infiltration of municipal authorities, coupled with interventions in the organisational architecture of urban bureaucracies. In contrast to earlier Structural Adjustment Programmes in which development actors enforced a clear package of reforms by holding national governments hostage to conditionality-laden policy measures, these modalities highlight how DFIs institutionalise change through subtle, vested and protracted mechanisms. To place them in the lineage of Structural Adjustment Programs indicates that MSA is a deeply political process, embedded in a political economy of structural domination.

## Introduction

‘Structural adjustment was never dead’, a former World Bank consultant told me in an interview and added that they would not ‘call it like this anymore and [would now] do it slightly differently’ (fieldnotes, 09.02.2020). This statement haunted me. In the wake of calls for trillions of dollars of annual infrastructure investment (CCFLA, 2023; World Bank, 2015), development initiatives have also aimed to build institutional conditions that facilitate the anchoring of such investment in cities. In this article I inquire into the ways in which climate-aligned development finance initiatives (DFIs) drive municipal reform. Under this label I summarise the programmes of development banks, multilateral organisations, development cooperations, global NGOs, city networks and philanthropies that seek to foster investment in cities, all justifying their efforts with a nod to climate change. Specifically, I explore the ways in which these initiatives build the legal-administrative grounds necessary to pursue this aim. To this end, I develop the notion of Municipal Structural Adjustment (MSA) as a lens through which to highlight the fields of intervention and modes of adjustment through which DFIs work.

To better understand processes of MSA, this paper engages with research on the legal-administrative underpinnings of climate-aligned development finance at the intersections of economic, urban and development geography (Lauermann, 2018; Mawdsley, 2016). In the subsequent section, I introduce research that has traced DFIs and the regulatory transformations that they promote under a green guise (Bigger and Webber, 2021; Webber et al., 2021). Yet, existing scholarly work – often focused more on emerging discourses and policy programmes (Long, 2021) as well as the financial flows themselves (Bernards, 2024) – could profit from further attention to the institutional transformations that are planned and advanced through DFIs. This focus not only allows for a better understanding of how transformations are entangled in and differ from previous waves of reform; it also provides an indication of how these transformations are rolled out in urban governance processes. Clearly, DFIs cannot hold cities hostage through the conditionalities of Structural Adjustment Programs. How, then, do they enforce urban governance reform?

The analysis I offer to answer this question and develop the notion of MSA focuses on Mexican municipalities and relies on a critical institutionalist perspective (May and Nölke, 2015). As I elaborate in the third section, this lens places attention on processes of institutional change, thereby accounting for actors that influence institutional work beyond formal spheres of governance, as well as for small-scale mechanisms and diverse power modalities advanced in this process. The argument I develop on this basis has four steps, each employing different data sets: First, in the fourth section, I build on an exploration of the ways in which DFIs impinged on municipal development during and in the aftermath of the policy era known as the Washington Consensus. Based on a review of secondary literature, I highlight the changing role of municipalities in such development interventions. While cities were also affected by Structural Adjustment Programs, I indicate that these development interventions were channelled through national-level institutions. Second, in the fifth section, I turn to more recent development efforts to show how cities have come to be a strategic target and terrain of development finance. Drawing on an analysis of DFIs in Mexico, I sketch an inventory of their fields of intervention, that is, the realm of municipal transformation they target. Rather than directly addressing governance structures, I argue that urban reform is addressed implicitly while interventions focus on the development of urban projects and sustainability strategies, the introduction of financial tools, as well as the production of urban data. Third, the sixth section contextualises these DFIs in the institutional context of Mexican cities, stressing municipal limitations with regard to political agency as well as to financial and fiscal capacity. Fourth, in the seventh section, the empirical core of this paper, I trace some of the DFIs’ local efforts in three secondary cities – Ciudad Juárez, Naucalpan and Hermosillo – to understand how MSA functions locally.

I delineate three interrelated modalities of adjustment that I take to characterise MSA: (1) the superimposition of DFIs’ planning schemes with government legislation and their gradual institutionalisation; (2) the burial of decisions in the development of infrastructure deals that come to define urban governance covertly ‘like a Trojan horse’ (interview, 17.01.2020) and (3) the infiltration of municipal authorities, coupled with interventions in the organisational architecture of urban bureaucracies. Together, these modalities highlight how DFIs institutionalise change through subtle, vested and protracted mechanisms. Unlike earlier Structural Adjustment Programs, in which development actors openly communicated and directly enforced a package of reforms, cities are offered support and participate in these programs on a voluntary basis. Yet agreed spheres of influence are slowly expanded, as the DFIs become interwoven in municipal decision-making. The covert nature of MSA heightens the need to revisit concerns raised in debates about Structural Adjustment Programs that are frequently overlooked in academic critiques of the economic violence of ‘green’ investment in the global South. To place MSA in the lineage of Structural Adjustment Programs not only highlights that MSA is founded in a global history of institutional domination but also underscores that local governance procedures are truncated and bypassed despite official discourse of municipal capacitation.

## Literature review: The urban as a site of climate-aligned development finance

How DFIs seek to adjust municipal governance is usefully contextualised in debates that discuss how the financialisation of development is operationalised in urban institutional interventions, for instance through the DFIs in focus here. My aim is to establish the grounds for a better understanding of these institutional transformations: the global context of development finance in which they emerge and the ways in which they are implemented in municipalities of the global South. I focus on the subfield of climate-related development finance rather than climate or development finance in general and, accordingly, foreground development finance debates. To be sure, both debates frequently overlap, as climate has been ‘mainstreamed’ in development interventions (Webber, 2016). Yet, as Bryant and Webber (2024) put it, climate finance ‘encompasses but far exceeds’ (p. 4) the DFIs in focus in this project, with the latter excluding, for instance, ESG-aligned real estate finance.

Debates in development finance (Mawdsley, 2016; Taggart and Power, 2024; Van Waeyenberge, 2016) document the ‘deepening union’ (Mader, 2015) between development finance and mainstream financial markets (Bernards, 2024; Mader, 2024; Mawdsley, 2018). Gabor (2021) helpfully explains these transformations through what she calls the ‘Wall Street Consensus’. Following Gabor, development institutions push ‘a hyper-financialised agenda’ (Carroll and Jarvis, 2022: 13) to adapt their interventions to the needs of portfolio investors (Gabor, 2021: 429) and mobilise the ‘trillions’ the World Bank has identified as lacking in regard to financing climate adaptation (World Bank, 2015). As part of this effort to close this ‘gap’ – so Gabor’s argument continues – the Wall Street Consensus implies that state institutions take a more active stance to de-risk economies, infrastructures and cities. Related interventions at the core of the DFIs I examine encompass attempts to foster private investment through project pipelines, data production and capacity-building, amongst others.

While Gabor’s intervention has led to a burgeoning debate that explores the increasing influence of private financial interests in development (Schindler et al., 2023), others have observed a lack of interest on the part of private investors (Bryant and Webber, 2024). To date, the World Bank’s target of trillions of dollars in investment fails to be met (Bernards, 2024; Dafermos et al., 2021). The sector itself explains this hesitation through what is variably termed the ‘capacity gap’ (Pinto, 2017) or the ‘project preparation gap’ (Farley et al., 2014) – the lack of frameworks, guidelines and administrative capacities that is seen to hinder investment. In following efforts to create the institutional underpinnings to overcome these hurdles, this paper centres on the institutional processes that anticipate financial investment, rather than the actual flows of capital or the financialisation of urban interventions.

To understand how these gaps are addressed in the messy policy processes of local institutions, it is useful to complement the insights of development finance with a body of research that has turned to understanding the functioning of DFIs at the urban scale (Bahadur and Tanner, 2014; Bhardwaj and Khosla, 2020). Scholars have examined the urban focus of development finance, philanthropic and UN level institutions, frequently contextualising these in rise of ‘the urban age’ (Acuto et al., 2023). Through analysing the urban programmes of these institutions – for instance, Rockefeller Foundation’s 100 Resilient Cities Initiative (100RC; Leitner et al., 2018; Madénian and Van Neste, 2023), or the Emerging and Sustainable Cities Initiative (ESCI) of the Inter-American Development Bank (IDB; Jiménez Corrales and Zagt Hernandez, 2022), which are also at the empirical core of this paper – this body of work indicates how global agendas are embedded locally to foster the financing of large urban infrastructure projects (Anguelov, 2023; Schindler and Kanai, 2018).

Some of this work has focused on the accompanying institutional transformations to show how municipalities have adapted their fiscal means (Goodfellow, 2018) or introduced financial tools (Herrera, 2024). Moreover, scholarship has examined the pivotal role of international financial and development institutions in the introduction of policy standards (Hilbrandt and Grubbauer, 2020), land reforms (Goldman, 2020) and fiscal changes (Cirolia, 2020), amongst other interventions in urban governance processes. A small number of scholars who are focused on the context of development finance understand these interventions in the context of structural adjustment. Bigger and Webber (2021) employ the terminology of green structural adjustment to address they ways in which DFIs provide for new opportunities to foster a re-engineering of policies and legal tools by coupling financial measures to presumably ecological ones. Baindur and Kamath (2009: 33) note processes of ‘municipal restructuring’ and illustrate how these foster the ‘dismantling of the public sector “monopoly” in municipal services and the entry of [the] private sector to improve these services’ (Baindur and Kamath, 2009), with crucial consequences for urban accountability. This paper shares that agenda. By adding an analysis of the processes at work to implement structural change at the urban scale, it moves our understanding beyond an analysis of policy programmes and their aims to delineate the specific *municipal* modes of structural adjustment, that is, ways of policy implementation as they play out in the messy political conditions of secondary cities. This analysis contributes a comprehension of how global agendas become institutionalised in municipalities that better attends to the everyday workings of the local state.

While not explicitly focused on development finance, I also draw on related bodies of work on subnational state finance and urban climate governance, as they provide detailed insights into some of the modalities through which global interventions are advanced in cities. The former body of work – studies on subnational state finance (Lauermann, 2018; Pike et al., 2019) – documents how municipal institutions are influenced by and experiment with finance and how these processes translate into legal-administrative change. Already in 1996, Sbragia (1996) described how local governments’ ‘Debt Wish’ drove financial experiments that changed the structure of municipal governments in the United States. Sbragia’s contributions and other work on finance-driven adaptations of urban institutions (Deruytter and Möller, 2020; Valverde, 2022) are insightful for understanding the role of municipalities in driving experiments with global finance. Yet, their predominant focus on European-American institutions makes these insights less apt for describing forms of policy interventions prevalent across the global South that are marked by the influence of development institutions, amongst other traits. The latter body of work – scholarship on urban climate change governance (Broto and Bulkeley, 2013; Bulkeley, 2012) – provides insights into the intersection of global politico-economic power and local institutional transformation: into the ways in which intermediaries implement governance change as cities become a testing ground for climate experiments (Bulkeley and Castán Broto, 2013). This paper builds on such work to advance insights into the institutional interventions that prepare the grounds for ‘green’ investment in Mexican secondary cities.

## Methodology: Towards an institutional analysis of DFIs

To understand how DFIs drive institutional change and develop the notion of MSA, I combine an analysis of empirical data on the programmes of DFIs with a case study approach that follows these programmes into selected cities, theorising that data through the lens of critical institutionalism. Specifically, I build on two data sets that are part of a larger study on the impact of global development finance on urban bureaucracies, conducted jointly with Erandi Barroso Olmedo and Fritz-Julius Grafe.

First, I rely on a document content analysis of the reports of the selected DFIs as well as on 35 expert interviews with development practitioners, experts, municipal officials, planners and consultants conducted between 2021 and 2023. DFIs were selected and documents chosen on the basis of their relevance for studying MSA. While DFIs are marked by differences between the diverse initiatives – that is, development banks (focused on selling loans), philanthropic organisations (which may provide grants) and development cooperation agencies (often focused on capacitation and sustainability concerns) – what they hold in common is their thematic focus on interventions in sustainable urban development and their aim to foster private investment. For this paper, I focused on initiatives active in Mexico, as I was familiar with and well networked in this context through prior field experience. Combined with interviews with the global and regional programme managers of these initiatives, this data provides key insights into the stated programme aims and strategies to implement them.

Second, the institutional focus of this paper entails following the programmes into the cities in which they engaged to study how their programmatic aims were implemented in place. The field sites – the municipalities of Naucalpan, Ciudad Juárez and Hermosillo – were chosen because they engaged with some of the DFIs we had studied. The expert interviews we conducted in-person and via zoom with municipal officials, planners and consultants were subsequently anonymised upon the request of these experts. Rather than an ethnographic exploration of these cities’ municipal transformations or an analysis of the socio-material outcomes of the interventions of select DFIs, I employ the data collected in these cities to sketch common identifiable features of regulatory interventions and discuss the ways in which they advance MSA.

To theorise this data, I build on a critical institutionalist analysis (Cleaver, 2012; May and Nölke, 2015) to highlight forms of adjustment that are protracted, vested and smaller in scale than the top-down reforms advanced through earlier Structural Adjustment Programmes. Critical institutionalism is designed to analyse how institutional structures stabilise and gradually change. My analysis of MSA is guided by three key elements that undergird this theory frame: First, in contrast to the ideals of rational-legal ‘Weberian’ state bureaucracies, critical institutionalism draws attention to how a wider set of institutions, actors at multiple scales and legal frameworks shape the institutional process (May and Nölke, 2015: 89): to soft or grey rules (including standards, local cultural norms and contracts) and to hybrid actor constellations (including development practitioners, global NGOs, local policy elites and financial players). Second, this approach brings, as Carstensen (2015) puts it, ‘agency-orientation to institutional analysis’ (p. 47). For critical institutionalists, political action and change emerge not only in larger disruption but also in everyday social practice (May and Nölke, 2015). Third, critical institutionalism goes beyond actor- and practice-centred theories in that it invites an investigation of the normative and cultural-cognitive dimensions of institutional change. Thereby, as May and Nölke (2015) note, ‘ideas and discourses should themselves be considered as institutions and therefore constitute subjects of critical institutional analysis’ (p. 91).

Two key implications of explicating MSA through this lens are worth highlighting: First, doing so widens definitions of the ‘structural’ aspects of adjustments to concern not only laws but also adjustments in normative and knowledge systems, as well as a wider range of informal rules (including contracts, standards and laws). Second, critical institutionalism puts modes of institutional transformation into view that lie beyond top-down interventionism, thereby echoing conceptualisations that account for the messiness and contested nature of urban governance. Using this lens thus allows me to inquire into the modalities through which political programmes are institutionalised. The subsequent sections trace processes of MSA through the interventions of DFIs and place them in the historical context of the Washington Consensus, to which I turn next.

## The municipality as a target of structural adjustment

Mexico was a poster child of Washington Consensus reforms. Following the government’s bankruptcy in 1982, its national institutions borrowed dollars through IMF loans (Toussaint, 2020). Similar to other international experiences, these loans were conditioned upon the prescriptive ‘policy package’ (Balassa, 1982) of Structural Adjustment, a 10-point programme that included reforms focused on fiscal discipline, privatisation, trade liberalisation and deregulation (Swaroop, 2016). Under the pressure of Structural Adjustment Programs’ conditionalities, Mexico oriented its policies towards free trade, the deregulation of foreign investment and the privatisation of state companies. These policy agendas were institutionalised in two treaties: first, the General Agreement on Tariffs and Trade, to which the country acceded in 1987 (Garza, 1996), and second, the North American Free Trade Agreement with Canada and the United States, which was signed in 1992 and fortified Mexico’s role as an export nation and its dependency on the United States.

In the wake of Structural Adjustment Programs, Mexicans (and populations across the Americas) suffered unemployment, hunger and poverty, during what has come to be known as the ‘lost decade’. While the scope of this paper does not allow me to detail the urban history of these interventions, including this historical background allows me to think through contemporary interventions with the structural violence of earlier programmes in mind. Moreover, their analysis permits me to trace the diverse roles assigned to municipalities in these policies. This provides the grounds to tease out how MSA differs from Structural Adjustment Programs and subsequent reforms.

As Fox and Goodfellow (2016) note, ‘cities ceased to be a major development priority’ under Washington Consensus reforms (p. 241). Consequently, Structural Adjustment Programs have rarely been analysed regarding their influence on urban governance (but see, Hamza and Zetter, 1998; Harris and Fabricius, 1996). Yet, Structural Adjustment Programs also had a set of urban components and effected the governance of cities through national-level change. Let me return to the case of Mexico. In 1983, President Miguel de la Madrid implemented decentralising reforms in order to ease the administrative burden of the indebted state, amongst other goals. Yet as these efforts were not accompanied by the strengthening of municipal administrative capacities, these reforms were bound to remain ineffective. Despite a rhetoric of decentralisation, city-focused reforms were marginalised to prioritise politics of deregulation and free trade (Garza, 1999: 156; see also Cabrero, 2000; Grindle, 2009). During Salinas de Gortari’s government (1988–1994), both the crisis and neoliberal policies as well as the rollback of urban-focused policies continued, for instance through abolishing the key national bodies tasked with urban development (Garza, 1996, 1999: 159). Instead, policy programmes assigned local government a narrow role confined to day-to-day tasks (Garza, 1999).

Still, during this period, Structural Adjustment Programs crucially shaped urban geographies and urban governance through interventions in two policy fields. First, reforms promoted the construction and privatisation of the urban infrastructure that was necessary to support the newly deliberalising economy, although indirectly through national policy change (World Bank, 1991). For instance, the fortification of public-private partnership (PPP) laws since 1991 enabled, as Guarneros-Meza (2009) puts it, the production of ‘territories that (could) be economically productive and competitive with the global urban system’ (p. 470). Accompanied by the introduction of special economic zones, these policies had devastating effects on the rapid development of cities and exacerbated regional inequalities through the asymmetric concentration of power in only a few cities. Second, development interventions of this era crucially influenced municipal governance through interventions in housing. The deregulation of the state’s housing programmes, beginning in October 1992, initiated a process of financialisation (Salazar et al., 2022; Salinas Arreortua and Gómez Maturano, 2022). Boils (2004: 347) describes how the World Bank dictated housing programmes through credit contracts (Cadena, 2002; Soederberg, 2014). In the face of the subsequent ‘tequila crisis’ (1994–1995), policies that built the grounds for housing financialisation, such as the sale of domestic banks, were pushed with further force. Again: In both fields of transformation, the municipal level was influenced by Structural Adjustment Programs not through measures directly targeting subnational government bodies but by leveraging national-level change.

Under the Zedillo government (1995–2000), decentralisation efforts became more intensified and aligned with globally circulating strategies of new public management (Ward et al., 1999: 2). This agenda followed international recommendations (Giugale and Webb, 2000) in shifting responsibilities down from central to regional and municipal scales while simultaneously targeting bureaucratic inefficiencies (Guarneros-Meza and Geddes, 2010) and enhancing citizen participation (Cabrero, 2003). New public management was outside of the initial canon of Structural Adjustment Programs, but it resonated with their policy prescriptions in that it fortified neoliberal governance projects of austerity and entrepreneurialism. Considering the modalities in which these changes were advanced, it is evident that they worked not through credit dependency and conditionalities but through ‘recommendations’ that already speak of post-Washington Consensus reforms.

Post-Washington Consensus measures – typically dated to begin in the late 1990s – were still not explicitly packaged as an urban programme. But they brought cities to the forefront of development debates. Policy reforms of this era were defined by a shift to urban governance programmes that moved urban policy ideas towards a ‘more integrated approach across the physical environment, infrastructure networks, finance, and institutional and social activities’ (Goldman, 2005) and opened larger development funding streams for the widening of municipal capacity (Dodman and Satterthwaite, 2008). While frequently framed as a pro-poor agenda of good governance, critical development scholars understand that agenda to extend earlier policy prescriptions (Mader, 2017; Van Waeyenberge, 2006), pushing policies that, to use the phrasing of Mawdsley and Taggart (2022), ‘acted to embed neoliberal governmentalities more firmly and deeply into lives, livelihoods, economies and processes’ (p. 6).

While this agenda continued to focus on creating a regulatory environment at the national level, it included a greater emphasis on municipal capacity-building, particularly for the support of urban strategies for competitiveness, and the inclusion of civil society in neighbourhood upgrading (Freire and Stren, 2001: xxii). Notably, these development interventions were addressing municipalities as a direct interlocuter, in particular to create state-market synergies (Fox and Goodfellow, 2016: 243), moving closer to MSA, to which I now turn.

## Taking stock of contemporary reform interventions: Four fields of MSA

Contemporary development studies describe the 2010s through a further paradigmatic transformation of development assistance that I have introduced with Gabor’s (2021) terminology of the Wall Street Consensus. In Mexico, where financial investment has enjoyed state support through decades of neoliberal governance, this era still furthered finance-led capitalism (see also Bastian Duarte and Jairath, 2019; Marois, 2014; Martínez, 2022; Reis and de Oliveira, 2021). In the last two decades, cities have also gained greater attention through the increasing pace and scale of urbanisation, the UN declaration of the ‘urban age’, and an avalanche of urban policy programmes rolled out at global and local scales, most prominently in the context of Sustainable Development Goal 11, now explicitly addressing urban development. DFIs can be seen to emerge at the intersection of these two developments.

Tracing the efforts of DFIs provides a useful ground from which to understand the policy fields where DFIs advance institutional interventions (Bazbauers, 2022). In what follows, I direct my focus to those measures that directly target municipalities. As I will show, urban governance is no longer ‘a specific objective’ (interview, 17.01.2020), but ‘a means to an end’ (i.e. financing infrastructure), as an interviewee from the German development corporation GIZ (the Deutsche Gesellschaft für Internationale Zusammenarbeit (GIZ) GmbH) told us in frustration. ‘At the moment’, he added, ‘you can’t move a dollar with governance issues. [What we do] has to be visible (. . .). You have to be able to have photos taken of buses or bicycle lanes’ (interview, 17.01.2020). Nonetheless, I argue, reform efforts continue, but they take place more indirectly through other fields of intervention. Let me introduce these fields to take stock of key lines of action that stand out across the analysed DFIs:

First, DFIs such as the IDB’s Emerging and Sustainable Cities Initiative (ESCI), GIZ’s FELICITY (Financing Energy for Low-carbon Investment – Cities Advisory Facility) programme, the Latin America Investment Facility’s (LAIF) City Life initiative or the Climate Finance Leadership Alliance target planning and urban development policy as well as urban projects. For instance, the ESCI, an initiative launched in 2012 and wound up in 2019, funded diagnostic studies and the development of so-called ‘action plans’ in 71 cities across Latin America (Crespo et al., 2016: viii), including 8 secondary cities in Mexico (Davis et al., 2019: 71). Second, project pipelines such as the World Resource Institute’s initiative TheCityFix Labs (WRI, 2023), Bloomberg Philanthropies’ Global Mayors Challenge or the ICLEI – Local Governments for Sustainability Transformative Actions Program (TAP, 2021) intervene in urban development by supporting the development of green projects designed to attract capital flows. Consider, for instance, the TAP pipeline, a partnership between public and private stakeholders, including the C40 City Finance Facility, the European Investment Bank and Global Covenant of Mayors’s Global Climate City Challenge: TAP supports cities in developing ‘mature, robust and bankable’ (TAP, 2021) infrastructure projects by introducing standardised metrics, making these implementable and putting them on display to potential investors to solicit private-sector funding.

Second, initiatives target the institutional architecture of municipal agencies by supporting agencies’ development of sustainability strategies. The Rockefeller Foundation’s 100 Resilient Cities (100RC) programme provides a useful example, as it broke new ground by installing privately funded offices within city bureaucracies. While it is common in Mexico that development agencies work directly from within the administrative units of national government bodies (e.g. the German development cooperation GIZ has an office in the Ministry of Environment and Natural Resources), the 100RC programme placed a DFI representative within an urban administration. While promoted through the label of resiliency, this field of intervention includes attempts to ‘break silos’ and integrate different administrations, interventions in the structures of administrative units, and endeavours to institutionalise strategic partnerships.

Third, initiatives roll out strategies to reform cities’ financial and fiscal policies through the introduction of financial metrics and financial tools. This includes efforts to expand municipal frameworks for collecting taxes (e.g. for land taxation) or the introduction of financial tools (e.g. green municipal bonds). These reform efforts are evident, for instance, in the World Resource Institute’s TheCity Fix Labs (e.g. introducing tools for land value capture), the World Bank’s City Creditworthiness Initiative (intending to improve municipal credit ratings) and the IDB’s ESCI (with a component on building sub-national fiscal capacity). Although working with different approaches, these initiatives cohere around strengthening the financial leverage of cities, thus making them ‘better’ actors in financial markets.

Across these policy fields, DFIs intervene at the level of knowledge transfer and technical support, for instance, by providing expertise and data. Most DFIs have a capacity-building component. Some host conferences, forums or workshops. Others have educational programmes. Yet others produce data that undergirds policy decisions and facilitates planning. For instance, the World Bank’s Global Program for Resilient Housing produces and processes geospatial data on climate and earthquake risks that it overlays with data on housing quality (World Bank, 2022). The IDB’s aforementioned ESCI supports participating cities with large-scale diagnostics of urban development challenges, for which it funds external experts that analyse the participating cities’ urban development challenges (using their own standards). The World Bank’s City Creditworthiness Initiative collects data through an online self-assessment tool (Grubbauer and Hilbrandt, 2023). As scholars have long argued, building up this knowledge infrastructure – for example, through the development of toolkits (modelling tools), business reports and plans – shapes the rationalities of the participating actors as well as possible crisis solutions.

In line with Structural Adjustment Programs and their city-focused adjustment plans, the described interventions target the possibility of creating profitable investment landscapes. Yet, this inventory shows that these interventions differ from earlier Structural Adjustment Programs and their city-focused adjustment plans in the (policy) fields that are addressed. Although these interventions target cities as a strategic terrain in which structural measures can be imposed, they institutionalise structural municipal transformations in protracted, more indirect ways, to which I turn after introducing the institutional context in which these transformations play out.

## The structural conditions of Mexican secondary cities

Zooming in on the institutionalisation of these fields of intervention in Mexican secondary cities, let me foreground the governance power of these cities and the role of DFIs in contemporary Mexico. In Mexico, municipalities have traditionally had very little power (Cabrero, 2000). Given their limited possibility of taxation, they largely depend on national funding streams and intergovernmental transfers from the state. Particularly in secondary or small cities, the technical and administrative capacities of state and particularly municipal governance are very constrained, and the success of federal programmes for strengthening these has remained limited. Consequently, the development, maintenance and operation of infrastructure is a severe problem (Giugale and Webb, 2000) that the climate crisis has made ever more pressing.

More recent federal policies have further limited municipalities’ financial flexibility. From 2018 to 2024, Mexico’s ‘left’-labelled government has advanced rather contradictory policies. López Obrador’s so-called ‘Fourth Transformation’, the government agenda of his 6-year term, can be described through the increasing promotion of austerity measures and debt ceilings (Streule, 2023), whereby key federal transfer mechanisms (such as the funding stream labelled ‘23’ (Ramo 23)), previously used for funding state and city infrastructure, have been cut. Moreover, the repurposing of funds towards a few large strategy projects, such as the Tren Maya, has pushed a financialisation agenda and centralised resources (Casañas, 2021) while further restricting state and municipal borrowing. States with existing international debt saw their loan management centralised under the Ministry of Finance. Today, municipal budgets depend heavily on allocations from this ministry as well as on property tax revenue. This dependency has constrained municipalities’ ability to access international funds, including for green projects.

At the same time, Obrador’s government has been challenging investment conditions through a nationalist discourse translated into policy, for instance, through changes to the Electricity and Hydrocarbons Laws (SEGOB, 2021) that promote energy self-sufficiency and have created uncertainty and, as I was told, prevented global private investment (interview, 09.02.2023). There is wide agreement amongst critics speaking from different sides of the political spectrum that neither of these measures is ecological and that they are imposed through opaque, undemocratic and populist measures (Ceceña and Ornelas, 2024; McElvain, 2023).

Though sustainable urban transformation may not be a national priority, cities – where climate change is most acutely felt – must find innovative ways to operate within these constraints. In the context of republican austerity, DFIs thus frequently constitute a rare possibility, which municipal officials may strategically employ to finance studies, gain capacities and leverage other forms of ‘support’ without much critical questioning (Hilbrandt, 2025). On the other hand, working with cities also causes development finance to be faced with numerous challenges, as our interlocutors explained at length, noting cities’ poor credit ratings, the longstanding connections between development agencies and national (rather than municipal) finance departments, the internal limitations of development agencies that prohibit them from financing municipalities and the absence of sub-sovereign lending schemes (interview, 17.01.2020). How, then, are reform efforts working with these constraints?

## Modes of institutionalising MSA

I forefront three modalities of MSA to discuss how DFIs intervene in cities: (1) the superimposition of DFIs’ planning schemes with government legislation and their gradual institutionalisation; (2) the burial of decisions in the development of infrastructure deals that come to define urban governance covertly, ‘like a Trojan horse’ (interview, 17.01.2020) and (3) the infiltration of municipal authorities, coupled with interventions in the organisational architecture of urban bureaucracies.

### The superimposition of planning schemes

When scrutinised about the IDB’s ESCI, our interviewees stressed that its services were purely voluntary: ‘The IDB comes to town and says, “if you want it, we’ll do it for you.” Of course, every city says yes [. . .] Everyone says “yes”’ (interview, 13.09.2021). To recall, the ESCI funded diagnostics and developed ‘action plans’, amongst others, in one of our case cities, Hermosillo. Saying ‘yes’ implied support through technical assistance, that is, international experts visiting the participating cities and providing a large-scale data analysis. Key to why local governments may strategically engage with development institutions in this way are the promises of expertise and of private investment, both of which are hard to conceive under Mexican austerity governance. Using their McKinsey-developed methodology with predefined indicators and related prioritisations, expensive international consulting firms, such as the multinational corporation IDOM, delivered studies including those conducted through co-creative tools (interview, 13.09.2021).

The IDB promoted the ESCI initiative as aiming to address the lack of comprehensive planning frameworks in many Latin American cities. While this rationale was certainly set in earnest, interviewed expert planners described additional economic motives behind these plans that were less openly communicated: ‘The idea is to sell one’s own loans for the projects that these studies make appear self-evident and urgently necessary’ (interviews, 13.09.2021; 19.02.2023). Rather than as an effort in integrated planning, action plans can also be seen as a portfolio of projects – an expanded asset inventory. Nonetheless, interviewees frequently described the initiative as a ‘toothless tiger’ or ‘much ado about nothing’ (interview, 09.02.2023). Despite having raised high expectations in cities and spent significant budgets on consultancy costs, financing the anticipated projects rarely became a realistic possibility (Crespo et al., 2016). As I was told with no lack of irony, the plans ‘remained as very nice documents in the offices of the mayors’ (interview, 09.02.2023).

Yet, thinking through these plans through the lens of MSA – that is, zooming in on the reform changes promoted in municipalities – shows these plans in a different light. In legal terms, action plans were non-binding frameworks with a guiding function only. In Hermosillo, they were employed as addendums to extant urban development plans, interlocked with the city’s existing plans by referencing projects that the administration had already lined up – an approach that Bhardwaj and Khosla (2020) describe as ‘superimposition’. Yet, despite the short-lived nature of the DFI and experts’ evaluation of its efforts as ‘toothless’, in Hermosillo, the ESCI’s action plans shifted the future planning trajectory. For instance, the ESCI’s propositions were taken up in subsequent planning schemes, as an expert from the World Resource Institute explained: ‘in the elaboration of the next [municipal] urban development program the [thus proposed] land management instruments were included’ (interview, 14.03.2023). Projects that had first been proposed in the action plan were later integrated in the urban development programme for the centre of Hermosillo. Follow-up plans piggybacked directly onto the IDB-paid plans – which had gone unnoticed or been written off as ‘much ado about nothing’ – and institutionalised what the IDB initiative had suggested. Speaking of the intentionality of this approach, the expert concluded that this was precisely what the IDB had purported ‘regarding how to influence public policy’ (Bhardwaj and Khosla, 2020). Although such work exceeds the scope of this analysis, it is possible to trace standards and guidelines developed out of these projects in the norms subsequently used at upper scales of government and in other municipalities (interview, 19.12.2022).

As a mode of MSA, this approach can usefully be described as the superimposition of DFIs’ planning schemes with government legislation and their gradual institutionalisation. In this way, DFIs foster MSA by adding non-binding plans by way of suggestion. As local governments assemble their policies, projects and ideas in a bricolage kind of way (Cleaver, 2002), they may draw on these plans and institutionalise DFIs’ policies in more binding ways into the ‘normal’ workings of governance.

### The burial of decisions in the development of infrastructure deals

Considering project pipelines – a central component of many city-focused development schemes – through the lens of MSA points to a similarly vested and protracted mode of MSA that DFIs advance: the burial of decisions in the development of infrastructure deals. As noted, project pipelines have replaced more integrated policies of good governance and decentralisation that were central to post-Washington Consensus reforms. Projects may, as indicated in the third section, frequently fail to be financed. Still, project deals advance MSA irrespective of the complications of bringing such deals about. As an expert from the City Finance Facility of C40 explained, structural change enters governance processes ‘a bit like a Trojan horse’: ‘Ultimately, the approach we hope to take is [. . .] not just a matter of ‘here’s the study, and the project is good now’, but that through the work on the concrete project we also look at what needs to improve in the city’ (interview, 12.02.2020).

The GIZ’s FELICITY pipeline, a programme co-funded by the European Investment Bank, provides a useful example of this approach. This pipeline serves to spark private-sector interest in green infrastructure projects, and, following the programme’s official announcement, ‘to support the design and structuring of low-carbon infrastructure projects from the perspective of international financiers’ (GIZ, n.d.). To this end, FELICITY offers technical assistance in the maturation of infrastructure concepts, while raising their legitimacy and visibility. In this development process, consultants introduce sustainability and accountancy standards, educate local experts and offer support in structuring deals (interview, 03.09.2021). Notably, and as one interviewee also pointed out, this and other DFIs are not actually, ‘giving the city a cheque for a million dollars’ (interview, 19.12.2022). Rather, DFIs select and commission consultancies and provide the city with the resulting report, keeping notable leverage in the process.

One featured project of this pipeline is an organic waste project in the municipality of Naucalpan that is neighbouring Mexico City. Naucalpan is developing this solid waste treatment plan together with FELICITY as well as with a considerable number of other international agencies.^1^ Based on the public-private partnership law introduced above, and through the support of financing mechanism called the National Infrastructure Fund (Fondo Nacional de Infraestructura; FONADIN), at the time of writing, these partners hoped to attract $39 million USD, matched by $16 million USD state investment (Banobras, n.d.).^2^ Additionally, to de-risk the project, Mexico’s National Bank BANOBRAS provided a grant to finance 50% of the project’s costs. Beyond outright economic motives, well captured by the Wall Street Consensus debate, the initiative aimed to institutionalise change through a process in which governance itself takes place in preparation of and through the project deal (Pike et al., 2019; Valverde, 2022). This process is usefully labelled ‘financing as governance’ – terminology that Johns (2011) employs to place greater attention on how ‘regulatory decision-making may be shaped by technical practices of financial deal-making, financial modelling and related re-configurations of institutional or jurisdictional space’ (pp. 391–392). As per Johns, project deals provide ‘public decision-making authority to private sector “deal specialists”’. Yet, the legal technologies used in those deals are already ‘littered’, as Johns (2011) suggests, with ‘decisions that flow from them with an autonomous logic that insulates them from particular types of inquiry while favouring other lines of inquiry’ (p. 393). Differently put, deals may not seem to have an impact on wider governance issues, but they restructure municipal work beyond the deal itself through assumptions and technicalities firmly embedded in deals.

Similarly, in the Naucalpan waste treatment plant, project planning has become entwined with the financial process, as the DFIs’ support was thinkable only in terms of a PPP deal, thus opening this infrastructure to the investment of private providers. Furthermore, the FONADIN credit line from BANOBRAS determined the revenue scheme, thus the city’s financial budget and tipping fees, echoing Johns’s concerns that deals entail approaches to decision-making that are seeing neither ‘like a state’ nor ‘like a city’ but rather like ‘a deal-maker, deal-seller or deal-consumer’ (Johns, 2011: 398). In this way, deal decisions are naturalised and governance officials tricked into seeing the world in a particular limited way. Notably, the structure of the deal determined fundamental decisions about the project. This is particularly true as the project development outlived multiple administrations, anchored in MOUs to secure its continuation. As described elsewhere (Hilbrandt and Grafe, 2023), initiatives thereby put coercive force on new incoming city governments to maintain support for these projects.

In distinction to Structural Adjustment Programs, project deals do not directly target spheres of governance. Rather, this modality of institutionalising MSA must be understood through the legal and financial technicalities of deal structures that shape lasting governance decisions in invisible ways. Unlike the image of the Trojan Horse our interviewee proposed, deals do not advance governance through subterfuge – intensions are not intentionally concealed. Yet, the real governance decisions become buried throughout the deal-making process, as DFIs retain control over the agenda.

### The infiltration of municipal administrations

Central to understanding the modes through which MSA becomes implemented is the DFIs’ infiltration of core governance functions, coupled, at times, with shifts in the organisational architecture of urban bureaucracies. The 100RC campaign provides a useful example. Through funding a ‘resilience office’ in multiple cities, this DFI was able to install key personnel directly within city government. For instance, the municipality of Ciudad Juárez applied to and entered the programme in 2014 (interview, 10.11.2022). The Rockefeller Foundation itself employed what they called a ‘director of resilience’ from existing government staff and paid them throughout the initial stage of the programme. Creating further dependency, they provided her with training, from which they then created a resilience strategy for the municipality. The Resilience Coordination office, which they subsequently worked to institutionalise, was founded in October 2018 with a twofold mandate: to implement the resilience strategy and to ‘strengthen international relations’ (interview, 10.11.2022). Our interlocutors described the approach as ‘a resounding failure in terms of resource allocation’, since the foundation decided to end the programme in 2019. Having installed the Resilience Coordination office, the director of resilience continued their work on the municipalities’ budget to advance the resilience strategy and the Rockefeller Foundation’s methodology (interview, 10.11.2022).

Through the lens of MSA, the 100RC campaign can be conceived of as the DFI’s takeover of core governance functions, thereby responding to a wider trend in DFIs’ interventions. We witnessed how DFIs contracted experts to take on the role of municipal planners. As an interviewee paid via one such contract explained to us, his job to design and elaborate public policy was in support, not in replacement, of public officers: ‘we are not the ones who develop it, but work as an accompaniment’ (interview, 14.03.2023). In practice, however, and considering the empirical realities of Mexican austerity governance, these actors become tasked to take on roles with decision-making mandates, that is, work they initially may not have intended to perform (interview, 11.02.2023). At the same time, consultants sent from philanthropies or development banks, such as the 100RC resilience officers, establish a hinge between municipal bodies, DFIs and, with that, development banks and consultancies. Working from within local government, they open the door to the further influence of these agencies to form coalitions of organized interest, thereby allowing the city to tap into further funding streams (interviews, 11.02.2023; 09.02.2023). The implementation of the Resilience Coordination and other similar interventions follow a city’s application. Yet, these measures are then slowly and covertly widened, thereby shifting municipal structures.

## Conclusion

Cities have long been the target of development reforms. Yet, placing DFIs in the context of historical interventions evidences that MSA differs in its fields of interventions and modes of interference from earlier development policies. Structural Adjustment Programs enforced a clear package of reforms that were implemented from the top down, as governments were held hostage by debt relations and subjected to ‘conditionality-laden’ policy measures. Their urban elements were implemented through national-level change; they did not directly address municipal bodies. In the contemporary interventions I labelled MSA, municipal institutions and their urban strategies themselves become the target of development interventions, less overtly and through indirect steps.

Three modalities of intervention are indicative of MSA: the superimposition of DFIs’ planning schemes with government legislation and their gradual institutionalisation; the burial of decisions in the development of infrastructure deals that come to define urban governance covertly; and, the infiltration of core governance functions, for instance, in the name of fostering resiliency. These are modalities of adjustment that proceed in subtle, protracted and vested ways. While targeted changes are, at first, not legally binding, they incrementally gain traction, as subsequent processes institutionalise these reforms. In this way, they advance a deeper integration of DFIs’ interests into the workings of the municipality. When cities are offered support on a voluntary basis, but agreed spheres of influence are slowly expanded and pre-made decisions are hidden behind instruments of co-design, urban administrative authority becomes diminished as DFIs are steering the state ‘from within’. As governance is here no longer ‘a specific objective’ (interview, 17.01.2020) but ‘a means to an end’ (i.e. financing infrastructure), interventions often go unnoticed.

The invisibility of MSA makes it ever more urgent to reiterate concerns loudly voiced in debates about Structural Adjustment Programs that are often left out of scholarly criticisms of infrastructural violence, profit extraction and the material flaws of these programmes. The lens of MSA can complement these accounts with a critique of development focused on their institutional interventions: of their interference in municipal autonomy and their lack of democratic accountability. MSA indicates that electorates are circumvented, governance procedures truncated and urban administrative authority diminished. While the processes of restructuring I draw into focus are contradictory, operate through what Goldman (2005: 12) calls the ‘more mundane’ type of ‘violence’, incompletely operationalised, and often invited by policy elites, to place them in the tradition of Structural Adjustment Programs indicates that MSA is a deeply political process, embedded in a political economy of structural domination.

## Notes

1. Including the European Investment Bank, U.S. Environmental Protection Agency’s Waste Initiative and Global Methane Initiative, the Global Methane Hub, German Reconstruction Credit Institute (KfW).

2. Fonadin, a national infrastructure trust fund, is a government agency administered by BANOBRAS (Banco Nacional de Obras y Servicios Públicos), a national development bank. It is co-financed through a mix of federal funds, loans from international development banks such as the World Bank, revenues from toll highways and private investment.
